# READ-COGvid: A Database From Reading and Media Habits During COVID-19 Confinement in Spain and Italy

**DOI:** 10.3389/fpsyg.2020.575241

**Published:** 2020-10-19

**Authors:** Ladislao Salmerón, Barbara Arfé, Vicenta Avila, Raquel Cerdán, Raquel De Sixte, Pablo Delgado, Inmaculada Fajardo, Antonio Ferrer, María García, Laura Gil, Nadina Gómez-Merino, Álvaro Jáñez, Gemma Lluch, Amelia Mañá, Lucia Mason, Federica Natalizi, Marina Pi-Ruano, Luis Ramos, Marta Ramos, Javier Roca, Eva Rosa, Javier Rosales, Alba Rubio, Marian Serrano-Mendizábal, Noemi Skrobiszewska, Cristina Vargas, Marta Vergara-Martínez, Manuel Perea

**Affiliations:** ^1^Estructura de Recerca Interdisciplinar Lectura, Universitat de València, Valencia, Spain; ^2^Università degli Studi di Padova, Padua, Italy; ^3^Universidad de Salamanca, Salamanca, Spain; ^4^Universidad Nebrija, Madrid, Spain

**Keywords:** reading habits, media habits, COVID-19, reading motivation, critical reading, distress

## Introduction

The COVID-19 outbreak severely hit the population of Europe in general, and Spain and Italy in particular. By 25th May 2020, both countries accounted for 17.3% of the COVID-19 related deaths and 8.5% of infections worldwide (EU, 2020). The severity of the situation at the beginning of March led their respective governments to pass highly restrictive laws that enforced strict confinement of the vast majority of the population. Within this context, we studied the way adults in Spain and Italy adapted their reading and media habits.

Several large studies on reading habits during adulthood have identified five main reading activities and goals: reading for leisure, reading study or work documents, reading news to keep up with current events, reading to socialize with others, and shared reading with children (see Scales and Rhee, 2001; Mol et al., 2008; Torppa et al., 2020). The study of reading habits in adulthood has brought extensive attention due to its relationship with psychological, emotional, and health conditions (see Marshall, 2020, for a review). Indeed, reading for leisure has a clear impact on adults' socio-cognitive well-being (Mumper and Gerrig, 2017). However, little is known about how those habits change and are affected by collective crisis in which the citizens are confined in their homes. One exception is the study of news reading habits during crisis. Extensive exposure to news related to community crisis (e.g., the 9/11 terrorist attacks) led to increased anxiety and non-adaptive health-protective and help-seeking behaviors (see Garfin et al., 2020, for a review). To the best our knowledge, no prior study has evaluated the changes in reading habits due to a collective crisis. A strict lockdown may impact people's free time available, which will set the ground for potential changes in reading habits. But such changes may depend on people's social (e.g., living alone or with minors) or individual characteristics (e.g., distress, reading motivations).

In the present paper, we present the READ-COGvid database, composed of responses of 4,800 individuals from Spain and Italy. While we focus on leisure and reading habits at different moments (before the confinement, shortly after confinement, and after 1 month confined), we also collected many other indices (*socio-demographic, psychological*, and *reading-related*) that may be of interest to researchers interested in adults' reading and related areas (e.g., communication research, cognitive sciences, social studies, health sciences, cross-cultural studies). The READ-COGvid database is freely available to all users at: https://osf.io/24et3/?view_only=68613c73dd71499bbdadbad93d4ca79a.

## Method

The READ-COGvid survey was designed by the Spanish and Italian teams who author the manuscript. We first wrote and agreed upon a version in Spanish. Subsequently, three team-members with knowledge of both languages (two Italian and one Spanish), prepared the translation from Spanish into Italian using “equivalence” as the main translation technique. That is, we chose specific linguistic forms in Italian that carry the same intended meaning as in the original Spanish. For the items related to the distress scale, the translation procedure differs slightly, as we took the items directly from the standardized Spanish (Escrivá et al., 2004) and Italian versions (Albiero et al., 2006).

### Data Collection

The Spanish survey was launched on April 11th, 2020, 1 week after Spain reached its peak on COVID-19 deaths on April 3rd, 2020, and was closed on April 19th, 2020. The Italian survey was launched on April 15th, 2020 and was available until April 24th, 2020, 2 weeks after the peak of COVID-19 deaths which had occurred during the last week of March.

In both countries, data for the READ-COGvid reading habits survey was collected through non-probability sampling. In particular, an unrestricted self-selected survey was applied (Fielding et al., 2017). We used this strategy to facilitate the access to population who would have been difficult to contact otherwise during the lockdown. Specifically, we published a link to the survey on social media and sent links to the survey to educational associations, undergraduate students from several universities in Spain and Italy, and to friends and family members with requests to forward it.

Responses were collected via the tool LimeSurvey, and data was stored in the servers of the University of Valencia, following the GDPR Compliance. The study was designed following the ethical principles of the Declaration of Helsinki. Before their participation, participants were informed about the goals of the study and about the ethical guidelines followed in the design and data treatment. No data was collected that could allow a third party to identify the respondents' identity. Participants were requested to provide their consent to data analysis prior to participating in the study.

A total of 11,634 people in Spain and 2,175 in Italy opened the survey, but only 4,181 in Spain and 837 in Italy completed all the questions. Despite our best efforts, many potential respondents abandoned the online questionnaire before completing it in full. However, it should be noted that low response rates are usually found when using web surveys. For example, Manfreda et al. (2008) carried out a meta-analysis of 45 studies examining differences in the response rate between web surveys and other survey modes, and estimated that the average response rate in web surveys was ~11% lower. In any case, the particular response rate of a survey study is associated with a number of variables, such as the specific topic, the survey length, the provision of incentives, the use of a mixed-mode survey design, or the use of pre-notifications and reminders (e.g., Fan and Yan, 2010). Regarding our study, perhaps a shorter survey (participants took on average 13.8 min) or giving incentives for participation would have increased the completion rate.

From the completed reports, we excluded 69 cases in Spain and 12 in Italy who did not provide consent to analyze their data, as well as 87 cases who did not live in Spain and 20 who did not live in Italy. In addition, we also excluded 2 participants for showing an incoherent pattern of responses and 7 apparent duplicates in the Spanish sample (0 in Italy). After exclusions, the final sample was composed of 4,013 respondents for the Spanish survey and 805 for the Italian survey. As shown in Table 1, the final sample was largely female, middle aged, and well-educated.

**Table 1:** Descriptive statistics (N, gender, age range, occupation, and completed studies) of the Spanish and Italian samples.

			**Spanish sample**	**Italian sample**
*N*			4,013	805
Gender	Female		2,797 (69.70%)	615 (76.30%)
	Male		1,188 (29.60%)	186 (23.08%)
	Not reported		28 (0.70%)	4 (0.50%)
Age range	From 18 to 24 years		1,634 (40.72%)	221 (27.45%)
	From 25 to 34 years		686 (17.09%)	207 (25.72%)
	From 35 to 44 years		552 (13.76%)	92 (11.43%)
	From 45 to 54 years		570 (14.20%)	128 (15.90%)
	From 55 to 64 years		410 (10.22%)	101 (12.55%)
	From 65 years on		161 (4.01%)	56 (6.96%)
Occupation^a^				
	Students		1,796 (44.75%)	255 (31.68%)
	Workers		1,968 (49.04%)	439 (54.53%)
		Working outside home	232 (11.79%)	86 (19.59%)
		Working from home	944 (47.97%)	192 (43.73%)
		Cessation of working activity	371 (18.85%)	107 (24.37%)
		Home isolation^b^	17 (0.86%)	8 (1.82%)
		Other	43 (2.18%)	13 (2.96%)
		No response	361 (18.34%)	33 (7.52%)
	Unemployed		223 (5.56%)	50 (6.21%)
	Retired		206 (5.13%)	59 (7.33%)
	Other		29 (0.72%)	18 (2.23%)
Completed studies				
	Primary education		43 (1.07%)	1 (0.12%)
	Secondary education		1,906 (47.50%)	338 (41.99%)
	Undergraduate degree		1,274 (31.75%)	385 (47.83%)
	Master degree		541 (13.48%)	41 (5.09%)
	Ph.D. degree		243 (6.05%)	37 (4.60%)
	Other		6 (0.15%)	3 (0.37%)

*Non-weighted frequencies and percentages (in brackets) are shown*.

a *Participants could select several options for the question “Occupation” (e.g., some respondents identified themselves as student and worker). For the “Occupation” categories percentages are calculated using the total number of respondents, whereas for the “Worker” subcategories percentages are calculated using the total number of respondents identified as workers*.

b *Due to COVID-19 diagnosis or symptoms*.

Considering that a convenience sampling procedure was applied in the current survey and, hence, findings could not be directly generalized to the population (Fielding et al., 2017), post-stratification is recommended as the weighting adjustment method to account for misrepresented groups in the intended population. To that end, we calculated weighting values adjusted for age and gender. To do so, we first computed the population reference values (i.e., frequencies by age range and gender) as provided by the Spanish (Instituto Nacional de Estadística, 2020) and Italian (Istituto Nazionale di Statistica, 2020) National Institutes of Statistics. Second, we computed the observed frequencies by age range and gender in the READ-COGvid database for each country. Next, we computed the weight value as W = (n_p_ / n_s_) × (N_s_ / N_p_), where n_p_ and n_s_ are the frequencies by age range and gender in, respectively, the population and the sample, N_s_ is the sample size, and N_p_ is population size.

### Measures

#### Reading and Media Habits

Participants repeated the reading and media habits scales three times. First, they recall the last time when they had spent a few days at home (e.g., holiday weekend). Second, they recall the first 2 weeks of confinement. Finally, they reflected about the current period, after a few weeks of confinement had passed.

#### Reading Frequency

This scale assessed how much daily time participants typically spend on different reading activities: reading for leisure, reading for work or study, reading news to keep up with current events, and social reading (Scales and Rhee, 2001; Torppa et al., 2020). Participants who indicated that there was at least one offspring living at home were also asked how much time they devoted to read with their son/daughter (Mol et al., 2008). For each reading activity, they answered using the following scale: nothing, ~30 min a day, 1, 2, 3, 4 h a day.

#### Reading Medium

This scale was intended to measure the use of reading mediums. This scale was nested into the reading frequency questionnaire. For each type, participants were asked the extent to which they used paper and digital (computer, tablet, or cellphone) reading media. They answered using a 5-point Likert scale, from Never (1) to Always (5).

#### Television Viewing Frequency

This scale assessed the daily time devoted to television watching or video streaming. Specifically, we asked respondents how much daily time they devoted to watch series, films, documentaries, and other programs. For each type, participants responded using the scale used in the reading frequency questionnaire.

### Psychological Indices

#### Test of Perceived Reading Difficulties

This 4-item scale is conceived as an indicator of participants' perceived reading comprehension skills. Participants specified if they experienced comprehension difficulties while reading texts in different formats: journal magazines, operating instructions of home devices, administrative forms, and bus or metro's timetables or maps, in a 5-point Likert scale from “totally disagree” to “totally agree.” The scale was adapted from the original instrument validated in Danish (Elbro et al., 1995, 2011). The internal consistency of the scale was acceptable, Spanish sample: ω = 0.74, Italian sample: ω = 0.77 (Omega indices used in this study were run using a polychoric correlation matrix, Gadermann et al., 2012).

#### Disposition Toward Evaluating COVID-19 Sources

This 5-item scale was designed to capture respondents' disposition to evaluate information when reading about COVID-19 (cf. Bråten et al., 2018). Participants indicated the frequency in which they engaged in different evaluation behaviors when reading about COVID-19: attending to author's information, attending to source information, contrasting sources, discarding unreliable sources, and reading expert sources. Participants used a 5 point Likert scale, from Never (1) to Always (5). The internal consistency was good, Spanish sample: ω = 0.80, Italian sample: ω = 0.80.

#### On-Task Attention Difficulties

We measured respondent's perceived difficulties to stay focused on tasks by using two items from the Mind Wandering Questionnaire (MWQ) developed and validated by Mrazek et al. (2013). The MWQ consists of five Likert-scale items measuring respondent's tendency to mind-wandering. We chose two items that referred to broad situations: (1) “I have difficulty maintaining focus on simple and repetitive work,” and (2) “I do things without paying full attention.” Respondents answered referring to the three confinement periods assessed, using a 5-point Likert scale, from Never (1) to Always (5). Average reliability of this measure across the three moments of confinement was acceptable (Spanish sample: ω = 0.79, Italian sample: ω = 0.74).

#### Motivation Toward Reading

We adapted the SRQ-Reading Motivation questionnaire (de Naeghel et al., 2012), developed to capture two autonomous types of reading motivation (intrinsic regulation—e.g., “I read because I enjoy reading”—and identified regulation—e.g., “I read because I think reading is meaningful”), and two controlled types of reading motivation (introjected—e.g., “I read because I will feel guilty if I don't do it”—and external regulation—e.g., “I read because others oblige me to do so”). We selected 8 items from the original questionnaire that represent both extremes: 4 items of intrinsic motivation-autonomous reading motivation, and 4 items of extrinsic motivation—controlled reading motivation. Items were scored on a five-point Likert scale, ranging from 1 (completely disagree) to 5 (completely agree). The four-item subscales in our samples had good internal consistency (Spanish sample: ω = 0.89 and ω = 0.82 for internal and controlled-external motivation, respectively; Italian sample: ω = 0.88 and ω = 0.87 for internal and controlled-external motivation, respectively).

#### Distress

The personal distress (PD) subscale of the Interpersonal Reactivity Index (IRI) (Davis, 1980) was used to assess participants' distress during the lockdown. This is a 7 items 5-point Likert scale tapping feelings of anxiety and self-control in tense situations. Respondents rated statements such as “In emergency situations, I feel apprehensive and ill-at-ease” from 0 (does not describe me well) to 4 (describes me well). We used the validated adaptations in Spanish (Escrivá, et al., 2004) and Italian (Albiero et al., 2006). The PD scale showed good internal reliability (Spanish sample: ω = 0.82; Italian sample: ω = 0.82).

### Other Indices

#### Socio-Demographic

Participants reported their gender, age in years, postal code, highest level of education completed, and occupation. Those who indicated that were workers or self-employed, where asked about their employment situation during the lockdown.

#### Cohabitation

Participants reported: (a) number of people living at home; if (b) was higher than one, (c) number of offspring at home and their age, and (d) whether there were people with disabilities living at home.

#### Average Grade

Students were asked to report their average grade from the last semester or assessment period. The Spanish grading system uses a 0–10-point scale (5 pass), whereas the Italian system uses a 0 to 30–point scale (18–25 pass) in university and a 0–10-point scale (6–6.5 pass) in primary and secondary school.

#### Exclusive Personal Digital Device

Participants were asked whether they had or not a digital device for their exclusive use before confinement and ~1 month after the confinement.

#### News Media to Stay Informed About COVID-19

Participants indicated the extent to which they rely on different news media to stay informed about COVID-19, in a scale from Never (1) to Always (5).

#### An Application of the Database

We illustrate the type of analyses that can be performed with the READ-COGVid database by identifying changes in reading times. Recent evidence shows that adults gradually improved their health behavior during the first week of COVID-19 confinement (López-Bueno et al., 2020). Based on this, we expected that young adults would increase their reading times for leisure reading, as it can lead to cognitive, emotional, and health benefits (Marshall, 2020). On the contrary, we expected no change or a decreasing time for news reading, as this may help to diminish the negative emotional impact linked to the exposure of news about collective crisis (Garfin et al., 2020).

To examine this issue, we conducted linear mixed effects models using the *lmer* and *emmeans* packages in R (R Development Core Team, 2020) in a subsample of Spanish young adults between 18 and 29 years old (*n* = 2009). The fixed factors were Time (Before vs. Beginning vs. Month), Type of reading (Leisure vs. News vs. Socialize vs. Study/Work), and Gender (Female vs. Male). Subjects was the random effect in the model. The dependent variable was number of reading hours per day.

Results showed that women spent more time reading than men, *F*_(1, 22293.1)_ = 101.743, *p* < 0.001. The main effect of Time was significant, *F*_(2, 22293.1)_ = 245.2660, *p* < 0.001, as well as the main effect of Type of Reading, *F*_(2, 22293.1)_ = 2226.569, *p* < 0.001. As the three-way interaction was not significant, *F*_(6, 22293.1)_ = 1.97, *p* = 0.066, we proceeded to examine the significant two-way interactions.

Gender interacted with Time, *F*_(1, 22293.1)_ = 3.399, *p* = 0.033, reflecting that the advantage in reading time of women over men increased with time in confinement. Gender also interacted with Type of reading, *F*_(3, 22293.1)_ = 96.523, *p* < 0.001, reflecting that gender differences were more pronounced for socializing (*z* = 18.758, *p* < 0.001) than for the other reading categories (leisure: *z* = 3.098, *p* = 0.002; news: *z* = 3.864, *p* < 0.001; study/work, *z* = 4.641, *p* < 0.001).

Critically, the interaction between Time and Type of reading was also significant, *F*_(6, 22293.1)_ = 26.416, *p* < 0.001. This interaction revealed that confinement modified adults' reading habits differently.

### News

With the beginning of confinement, individuals tended to consume more news than before confinement (*z* = −8.288, *p* < 0.001), but this consume of news decreased to baseline level after 1 month (beginning vs. month: *z* = 7.063, *p* < 0.001; before vs. 1 month: *z* = −1.23, *p* = 0.434).

### Leisure

Early in the confinement, individuals tended to read more for leisure than before confinement (*z* = −14.082, *p* < 0.001) and this pattern was stable after 1 month (beginning vs. month: *z* = −0.445, *p* = 0.896; before vs. 1 month: *z* = −14.527, *p* < 0.001).

### Socialize

Individuals used social networks more frequently at the beginning of the confinement than before confinement (*z* = −10.869, *p* < 0.001). While the use of social networks decreased after 1 month of confinement relative to the beginning of confinement (*z* = 4.264, *p* < 0.001), it was still higher than the baseline level before confinement (*z* = −6.604, *p* < 0.001).

### Study/Work

**Figure 1:**
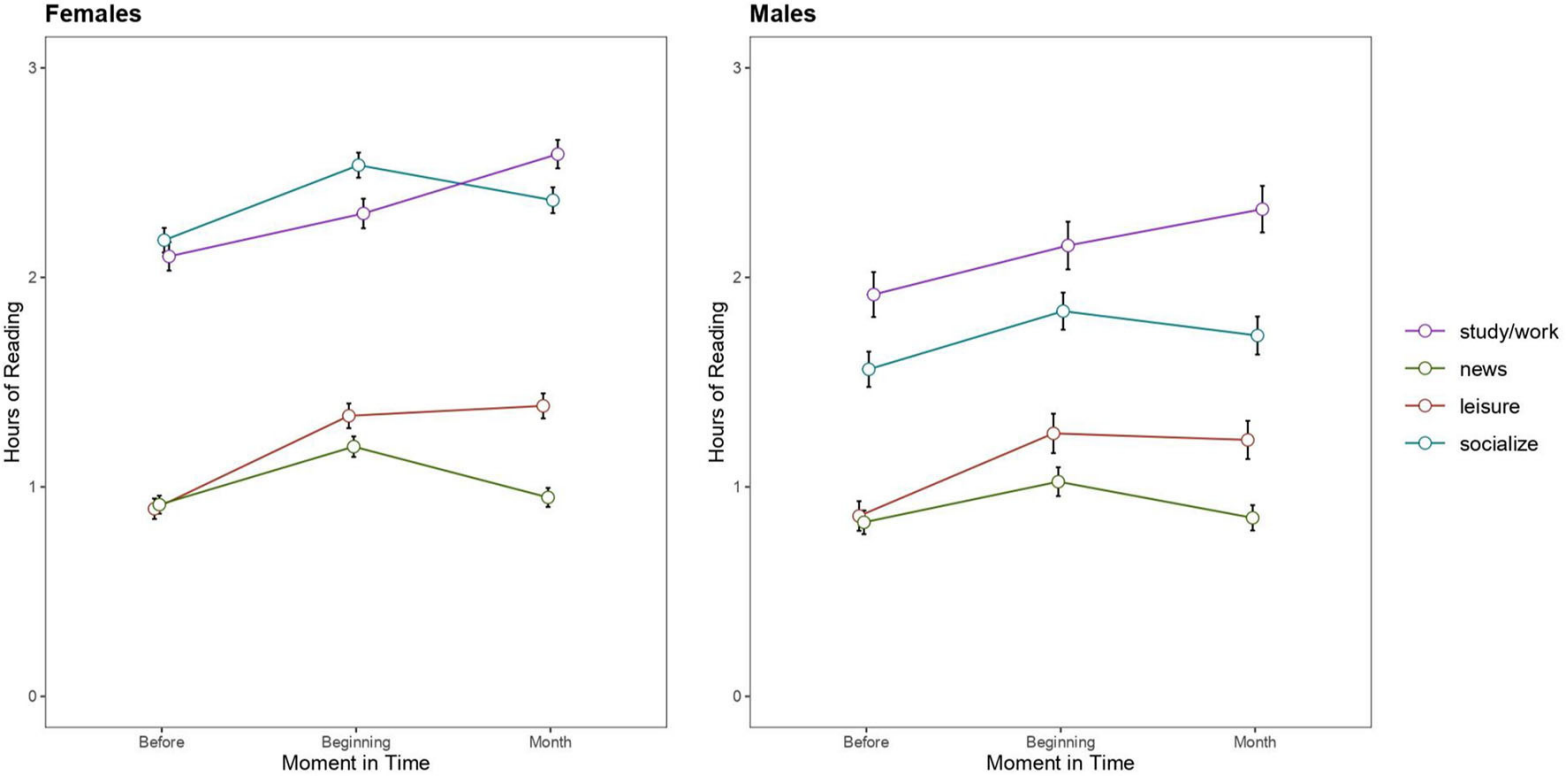
Mean and 95% confidence intervals of each condition for the dependent variable “number of hours reading”.

The time spent reading to study or for work increased at the beginning of confinement relative to baseline (*z* = −7.422, *p* < 0.001) and, in turn, increased after 1 month of confinement relatively to its beginning (*z* = −5.882, *p* < 0.001) (see Figure 1).

Immediate increases in all reading categories followed adaptive patterns as the confinement progressed. Relative to the initial 2 weeks of confinement, social and news reading decayed after 1 month of confinement, whereas leisure reading continued stable, and the time dedicated to reading for study/work continued to increase. Although these effects were independent of gender, women spent more time reading than men—this gender gap was more pronounced in the use of social networks (Tufekci, 2008).

## Conclusions

The READ-COGvid database provides a valuable tool about a unique period in which tens of millions of citizens remained confined at home in Spain and Italy. The large sample and the ample number and variety of indices allow researchers to design their own working hypotheses in the context of psychological and language sciences. Here we discuss two examples. First, our knowledge about reading habits is based on research looking at specific points in time. Factors such as television consumption, reading motivation, reading skills, gender, or age shape adults' reading habits (Scales and Rhee, 2001; Mokhtari et al., 2009; Applegate and Applegate, 2010; Locher and Pfost, 2019). Future research could use the READ-COGvid database to move forward the field by exploring to what extent those factors modulate the observed changes in reading habits during lockdown. Second, the study of critical reading has mostly focused on laboratory studies—these studies have been criticized because adopting a critical stance toward sources is a strategic behavior that may largely depend on the extent to which people perceive the situation as relevant for them (List and Alexander, 2017). Data from the READ-COGvid database provide information about peoples' critical attitudes toward source evaluation in a situation relevant for their lives, and this aspect could be studied in light of participants' distress, motivation and other contextual factors such as distance to COVID-19 epicenters (see Pennycook et al., 2020).

To sum up, by releasing the READ-COGvid database we expect to contribute to spur our knowledge on peoples' adaptation of reading and media habits during an unprecedented confinement period. Enriching such knowledge may help us to be better prepared to make informed recommendations in future similar situations.

## Data Availability Statement

The datasets generated for this study can be found in online repositories. The names of the repository/repositories and accession number(s) can be found in the article/ supplementary material.

## Ethics Statement

Ethical review and approval was not required for the study on human participants in accordance with the local legislation and institutional requirements. The patients/participants provided their written informed consent to participate in this study.

## Author Contributions

NS and MS-M implemented the study. PD, IF, MP-R, ER, JRoc, LS, and CV conducted the statistical analyses. BA, RC, PD, RD, IF, LM, MP-R, JRoc, LS, and CV drafted the manuscript. All authors performed substantial contributions to the conception of the study, reviewed the manuscript critically for relevant intellectual content, and approved the submitted version.

## Conflict of Interest

The authors declare that the research was conducted in the absence of any commercial or financial relationships that could be construed as a potential conflict of interest.
